# Increased Activity of a NK-Specific CAR-NK Framework Targeting CD3 and CD5 for T-Cell Leukemias

**DOI:** 10.3390/cancers14030524

**Published:** 2022-01-21

**Authors:** Elisaveta Voynova, Nga Hawk, Francis A. Flomerfelt, William G. Telford, Ronald E. Gress, Jennifer A. Kanakry, Damian Kovalovsky

**Affiliations:** Experimental Transplantation and Immunotherapy Branch, Center for Cancer Research, National Cancer Institute, NIH, Bethesda, MD 20892, USA; elisaveta.voynova@nih.gov (E.V.); voongn@mail.nih.gov (N.H.); flomerff@mail.nih.gov (F.A.F.); telfordw@mail.nih.gov (W.G.T.); gressr@exchange.nih.gov (R.E.G.); jennifer.kanakry@nih.gov (J.A.K.)

**Keywords:** CAR-NK, CAR-T, CD5, CD3, T-cell lymphoma

## Abstract

**Simple Summary:**

Chimeric antigen receptors (CAR) can redirect the activity of NK cells to target T-cell malignancies. Our results identify that recognition of CD5 molecules in malignant T cells by the CAR leads to improved antitumor response compared to targeting CD3, due to strong downregulation of the CD3 antigen after CD3-CAR treatment. We have also identified that a specific CAR-NK framework has superior activity than a CAR-T framework on NK effector cells.

**Abstract:**

NK effector cells expressing a CAR construct may be used to target T-lineage markers. In this work, we compared the activity of a NK-specific CAR-NK and a CAR-T framework when expressed on NK effector cells to target CD3 and CD5 in T-cell malignancies. Our results show that CD3-CAR-T is more active than CD5-CAR-T to eliminate malignant T cells in vitro, however, CD3-CAR-T were less efficient to eliminate tumor cells in vivo, while CD5-CAR-T had antitumor activity in a diffuse xenograft model. Lack of in vivo efficacy correlated with downregulation of CD3 levels in target T cells after coculture with CD3-CAR effector cells. The CAR-NK framework greatly improved the efficacy of CARs leading to increased degranulation, cytokine secretion and elimination of the tumor xenograft by CD5-CAR-NK effector cells. Finally, all CAR constructs were similarly effective to eliminate malignant T cells in vitro. Our results show that the NK-CAR framework improves the activity of CARs in NK cells and that CD5 would be a better target than CD3 for T-cell malignancies, as dynamic downregulation of target expression may affect in vivo efficacy.

## 1. Introduction

Recent years have shown remarkable success of chimeric antigen receptor (CAR) therapy for the treatment of advanced B-cell malignancies such as relapsed and refractory B-cell acute lymphoblastic leukemia (ALL) and B-cell non-Hodgkin lymphomas [1], bringing a possible cure to an otherwise untreatable condition. The effectiveness of these treatments relies on targeting the B-cell lineage marker CD19, which is shared by healthy and malignant B cells, leading to their elimination.

A similar advance has not yet been attained for the treatment of T-cell malignancies, mostly due to a lack of lineage markers that would be expressed on malignant and not on healthy effector T cells. Expression of the target molecule in CAR-T cells would result in a fratricide effect in which CAR-T cells eliminate CAR-T cells, leading to lack of efficacy.

Attempts to target T-cell markers with CAR-T cells for T-ALL have been tested. CAR-T cells specific for CD4 have shown reduction in xenograft tumors in NSG mice [2], and CAR-T cells targeting CD5 were shown to have a reduced fratricide effect that allowed an antitumoral response in a xenograft model [3]. However, CAR-T cells targeting CD5 had a fratricide effect that was more evident when the CAR contained the 4-1BB costimulatory domain [4].

To overcome these limitations, several groups are focusing on generating effector cells that would be resistant to fratricide for T-cell malignancies. One strategy to reduce the fratricide effect is to pharmacologically inhibit CAR expression in culture [4]. Another strategy is to inhibit the target expression, such as CD7 in CAR-T cells specific for CD7 by introduction of a protein expression blocker with the CAR. This blocker would bind to intracellular CD7 and retain it in the ER, leading to its absence in the cell surface [5]. TALEN-mediated disruption of the TCR/CD3 complex was required for the generation of CD3-specific CAR-T cells [6], and CRISPR-mediated deletion of CD7 allowed the generation of CD7-specific CAR-T cells for T-cell malignancies [7]. However, a possible drawback of these approaches for T-cell malignancies is that CAR-T cells are derived from the patient’s own T cells due to HLA restrictions. Therefore, the possibility of introducing the CAR into malignant T cells always exists, which may lead to uncontrolled proliferation driven by recognition of the antigen, as well as to significant toxicities.

Alternatively, natural killer (NK) effector cells may represent an off-the-shelf therapy, as they are not restricted to a specific HLA and can be redirected to target tumor cells by expression of a CAR. These cells do not express lineage markers present in T cells and could therefore be used to target T-cell malignancies without the risk of fratricide. The human NK92 cell line was also shown to be well-tolerated in clinical trials [8,9] and current trials are evaluating NK92-expressing CAR constructs for diverse antigens and tumor types (NCT02944162, NCT03383978, NCT03940833, NCT02892695, and NCT02742727) [10].

In this work we performed a comparative analysis of the efficacy of a CAR-T and a CAR-NK framework when expressed on NK cells to target CD3 and CD5 molecules on Jurkat and T-cell malignancies. 

## 2. Materials and Methods

### 2.1. CAR Construct Design and Characterization

To ensure correct localization of the CARs to the cell membrane, all CAR constructs contained the CD8 leader sequence (MALPVTALLLPLALLLHAARP) followed by a scFv derived from the OKT3 antibody to target CD3 or a scFv derived from H65 antibody to target CD5. The CARs with T framework contain the hinge region, transmembrane and costimulatory domains of CD28 and CD3ζ stimulatory cytoplasmic domain. The CARs with NK framework contain a CD8a hinge region, NKG2D transmembrane, 2B4 co-stimulatory domain and CD3ζ signaling domains. 

Retroviruses were produced using a 293GP packaging cell line by transient co-transfection with retroviral vector plasmid encoding the CAR-T and CAR-NK constructs that were generated in the laboratory (MSGV-CD3-CAR-T, MSGV-CD3-CAR-NK, MSGV-CD5-CAR-T, MSGV-CD5-CAR-NK) and a plasmid encoding the RD114 envelope protein. Culture supernatant containing retroviral particles was harvested after 48–72 h and stored at −80 °C. NK-92 cells were centrifuged at 32 °C for 2 h with viral particles onto retronectin (10μg/mL, Takara Bio, Mountain View, CA, USA)-coated multiwell plates. After transduction, NK-92 cells were expanded for 2 weeks in RPMI media. Presence of the CARs on the cell surface was determinate by FACS analysis of transduced NK-92 cell stained with biotinylated protein-L (Thermo Scientific, Waltham, MA, USA) followed by staining with Streptavidin PE antibody (Invitrogen). Protein L binds to the k subunit of single-chain antibody fragments (scFv) and Fab fragments. Protein L-positive cells were sorted using FACS Aria II (BD). NK92 cells were stably transduced to overexpress IL2 using (pAIP hIL2 co, Addgene, Watertown, MA, USA) plasmid [11]. 

### 2.2. Mice and Treatment

NOD-scid IL2Rgnull (NSG) mice were obtained from NCI/Frederick mouse facility and were maintained under pathogen-free conditions. All animal experiments were carried out in accordance with and approved by NCI ACUC protocol ETIB-015-2. 

Diffused tumor model: 8–12-week-old female NSG mice received intravenous administration of 1 × 10^6^ Jurkat cells that express luciferase on day 0. Followed by intravenous administration of 10 × 10^6^ CARs or control NK-92 cells on day 3 and 5 × 10^6^ CARs or control NK-92 cells on day 8. Each group contained 5 mice (*n* = 5). Mice were grouped by unmatched randomization. Evaluation of tumor infiltration was measured by average radiance read 10 min after i.p. injection of 200 μL of 15 mg/mL luciferin (Syd labs, MA, USA) in a Xenogen IVIS system (PerkinElmer, Hopkinton, MA, USA). Images were analyzed using Living Image Software (PerkinElmer, Waltham, MA, USA).

### 2.3. Flow Cytometry

Single cell suspension was processed and stained according to standard protocols. In brief, cell number was adjusted to a concentration of 1–5 × 10^6^ cells/mL in ice-cold FACS Buffer (PBS, 5% FBS, 5 mM EDTA). Then, cells were stained with labeled antibodies for 20 min at 4 °C, washed with FACS buffer and measured. The following antibodies were used: fluorochrome-conjugated monoclonal antibodies against human CD3-APC Cy7 (BioLegend, San Diego, CA, USA), CD5-BV421 (UCHT2, BioLegend, San Diego, CA, USA), CD7-PE (BD Pharmingen, Franklin lakes, NJ, USA), CD19-BV650 (HIB19, BioLegend, San Diego, CA, USA), CD56-APC (BD Pharmingen, Franklin lakes, NJ, USA), CD107a (Invitrogen, Waltham, MA, USA). For intracellular staining, cells were fixed and permeabilized with protein transport inhibitor Golgi Plug (BD, Franklin lakes, NJ, USA) and Monensin (eBioscience, San Diego, CA, USA). Cells were analyzed on a LSR Fortesa (BD Biosciences) using FACSDiva software v.9.0 (Franklin lakes, NJ, USA) Data were analyzed using FlowJo software v.10.7.1 (Tree Star, Ashland, OR, USA).

### 2.4. Patient Samples and Cell Lines

Human primary tumor samples were obtained from peripheral blood and bone marrow aspirate samples collected from patients with active disease in blood or marrow in clinical trial NCT03922724, a prospective clinical trial of allogeneic hematopoietic cell transplantation for peripheral T-cell lymphoma, approved by the Institutional Review Board. 

NK92 cell line, T-ALL Jurkat cell line and B-CLL MEC1 cells line were obtained from ATCC (Manassas, VA, USA) and cultured in RPMI1640 media (plus 10% FBS, 100 U/mL penicillin, 100 μg/mL streptomycin, and 2 mM L-glutamine). NK92 cells not transduced with IL-2 were cultured in the same media supplemented with IL-2 (100 IU/mL) with maintenance cell density of 0.3–1 × 10^6^ cells/mL.

### 2.5. Cytotoxic Assay

Specific cytotoxicity assays were carried as described previously [12]. Briefly, different NK92 CAR constructs were cocultured with target cell line Jurkat at the indicated effector: target ratios 1:1, 3:1 and 6:1. A reference (control) cell line MEC1 which does not express any target antigen was added to each well. Jurkat cells were labeled with CPD-eF450 and MEC1 cells with CFSE. Cells were cocultured for 4–5 h and analyzed by flow cytometry. Specific lysis was calculated using %specific lysis = [(negative target-experimental target)/negative target × 100]. 

Cytotoxic activity on primary leukemia cells was performed by coculture of unlabeled PBMC or bone marrow with NK92 effector cells at a 1:1 or 10:1 proportion between effector NK cells and target T cells. As different patients contained different numbers of target T cells including leukemia cells, before the cytotoxic experiment we identified the proportion of T cells by measuring the percentage of CD5 T cells in the patient samples. After coculture, cells were labelled with CD56, CD3, CD5, CD7 and CD19 to identify effector NK cells (CD56+), T cells (CD3+/CD5+), leukemia cells (CD5+ CD7‒) and primary B cells (CD19+). The number of primary B cells before and after coculture was used as reference for quantitation of cytotoxicity.

### 2.6. Cytokine Assay 

For in vitro IFNy cytokine secretion experiments, 5 × 10^5^ CARs cells and 5 × 10^5^ Jurkat target cells were cocultured for 16–24 h. ELISA was performed according to the IFNy kit instructions (R&D System).

### 2.7. Statistics

Unpaired student *t*-tests were used to determine significance of different groups. Tests with *p* < 0.05 were considered significant. Statistical analyses were performed using GraphPad Prism software (v10.0, GraphPad Software, La Jolla, CA, USA).

## 3. Results

### 3.1. Design of CAR Constructs

**Figure 1:**
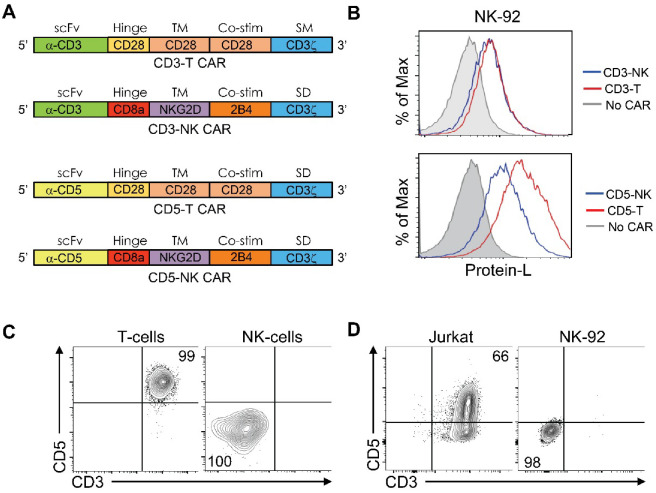
CARs construct design and characterization. (**A**) Schematic structure of the CD3 and CD5 CARs in a CAR-T framework (CD3-T and CD5-T) or in a CAR-NK framework (CD3-NK and CD5-NK). The identity of the different domains is represented—scFv, hinge, transmembrane (TM), co-stimulatory (Co-stim) and stimulatory (SM) domains. (**B**) FACS analysis of cell surface CAR levels in transduced NK-92 cells stained with Protein-L. (**C**) FACS analysis of target molecules on primary cells. T cells correspond to CD3+ and NK cells correspond to CD56+ PBMCs from healthy donors. (**D**) FACS analysis of target molecules on cell lines. Data are representative of two independent experiments.

To analyze the activity of a CAR-T and CAR-NK framework when expressed in NK effector cells, we designed CAR-T and CAR-NK constructs recognizing human CD3 and CD5 in T cells. The scFv recognizing CD3 was derived from the sequence of mouse OKT3 antibody [6] and the scFv recognizing CD5 was derived from the sequence of the humanized H65 antibody [13]. The CAR-T framework contains the hinge region, transmembrane and costimulatory domains of CD28, while the CAR-NK framework was derived from proteins expressed on NK cells and corresponded to a CD8a hinge region, a NKG2D transmembrane and a 2B4 costimulatory domain (Figure 1A) [14]. We generated stable human NK92 cell lines expressing these constructs and corroborated the presence of the CARs on the cell surface by protein-L staining, which recognizes the Kappa subunit of the scFv, and FACS analysis. We observed that all CAR constructs were correctly expressed and could be detected on the cell surface of NK cells (Figure 1B). FACS analysis confirmed absence of expression of the target molecules on primary NK cells and the NK92 cell line, and their presence on primary T cells and the T-ALL cell line Jurkat (Figure 1C,D).

### 3.2. Activity of the CAR Constructs In Vitro

**Figure 2:**
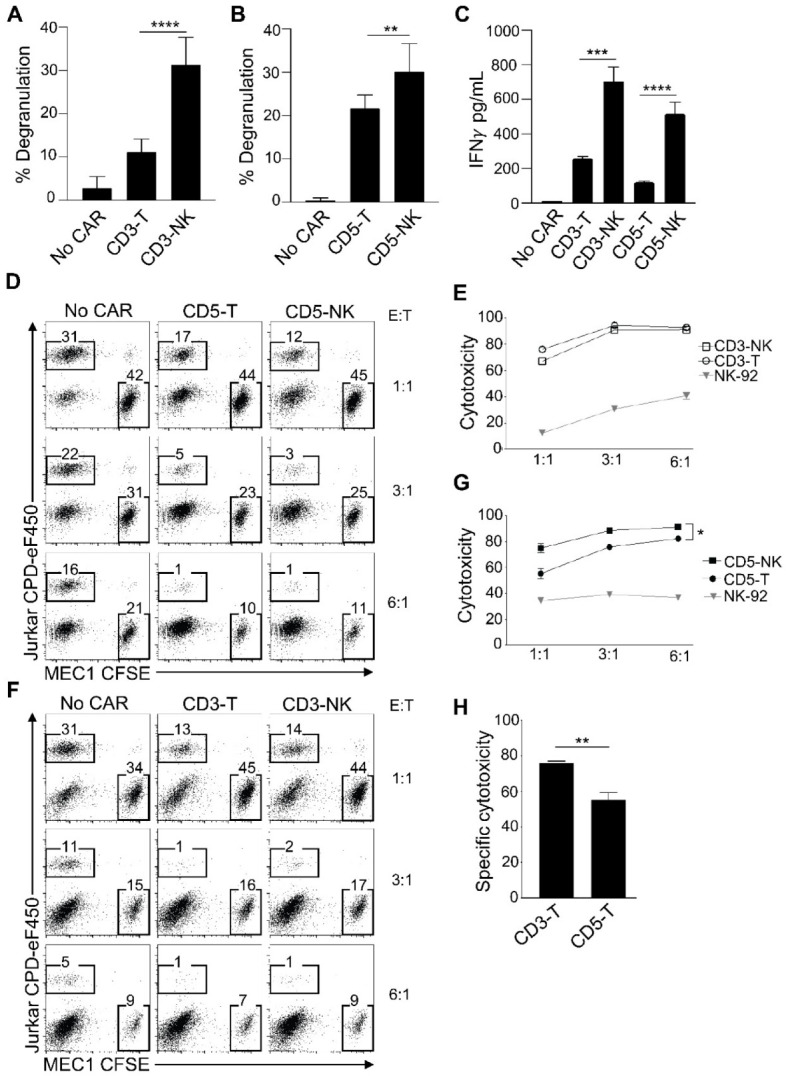
Effector function of CAR NK cells. (**A**,**B**) Quantitation of the percentage of effector NK cells that degranulated and became CD107+ after coculture with target cells at an effector: target ratio of 1:1. The graphs correspond to three independent experiments performed in quadruplicates. (**C**) Analysis of IFNγ secretion to 200 μL of the cell supernatant after coculture of 5 × 10^4^ CAR- NK cells with target cells for 16–24 h. Data correspond to three independent experiments. Unpaired *t*-test, *n* = 3. *p* < 0.05 (*), *p* < 0.01 (**), *p* <0.001 (***), *p* < 0.0001 (****). (**D**,**F**) In vitro cytotoxic assay after incubation of indicated CAR NK effector cells with target cells (Jurkat) and control cell line (MEC1) for 4–5 h at the indicated effector: target ratios. These graphs represent one of three independent experiments. Quantification of cytotoxicity at the indicated effector: target cell ratios of CD3 CARs (**E**) and CD5 CARs (**G**). One-way ANOVA test, *n* = 3, *p* < 0.05. (**H**) Specific cytotoxic comparison between CD3-CAR and CD5-CAR with T framework. Unpaired *t*-test, *n* = 3, *p* < 0.01 (**).

We next evaluated the activity of these CARs to recognize the Jurkat T-ALL cell line and trigger degranulation, measured as upregulation of CD107, a marker of cytotoxic activity. Coculture of CD3-CAR-T and CD5-CAR-T NK cells with Jurkat cells led to an increase in degranulation, represented as a significantly increased proportion of cells presenting CD107-positive staining compared to NK cells not expressing a CAR. NK cells expressing CD3-CAR-NK and CD5-CAR-NK had significantly increased degranulation when compared to CD3-CAR-T and CD5-CAR-T, respectively (Figure 2A,B). Analysis of IFN-γ cytokine secretion by ELISA after coculture with target cells showed that all CARs led to increased IFN-γ levels by NK cells, and this secretion was significantly increased in cells expressing the CD3-CAR-NK and CD5-CAR-NK constructs (Figure 2C), indicating a superior activity of the CAR-NK framework. 

We next analyzed the cytotoxic activity of these CARs to eliminate leukemia cells in vitro. We cocultured NK effector cells with a 1:1 mixture of carboxyfluorescein diacetate, succinimidyl ester (CFSE)-labeled negative control MEC1 B cells and cell proliferation dye (CPD)-labeled target Jurkat cells. Identification of remaining target cells was performed by FACS analysis. We observed a reduction in the CPD-to-CFSE-labeled cells ratio after CAR treatment, indicating that NK effector cells expressing all CARs efficiently targeted Jurkat cells and not the nontarget MEC1 cells at different effector-to-target ratios (Figure 2D,F). Quantitation of these data showed that CD3-CAR-T and CD3-CAR-NK had similarly high cytotoxic activity (Figure 2E) and CD5-CAR-NK had significantly higher cytotoxicity than CD5-CAR-T (Figure 2G). Interestingly, the cytotoxic activity of CD3-CAR-T was significantly higher than that of CD5-CAR-T at a 1:1 effector to target ratio (Figure 2H), and as shown in Figure 2E the cytotoxic activity of CD3-CAR was not improved by the CAR-NK framework at effector-to-target ratios of one or greater. At effector-to-target ratios lower than one, an increased efficacy of the CAR-NK framework could also be observed in CD3-CARs (Supplemental Figure S1). Cytotoxic activity was also observed as a specific increase in apoptotic target cells (Supplemental Figure S1). 

### 3.3. Efficacy of CAR Constructs to Eliminate Peripheral T-Cell Lymphoma and Downregulation of the Target Antigen

**Figure 3:**
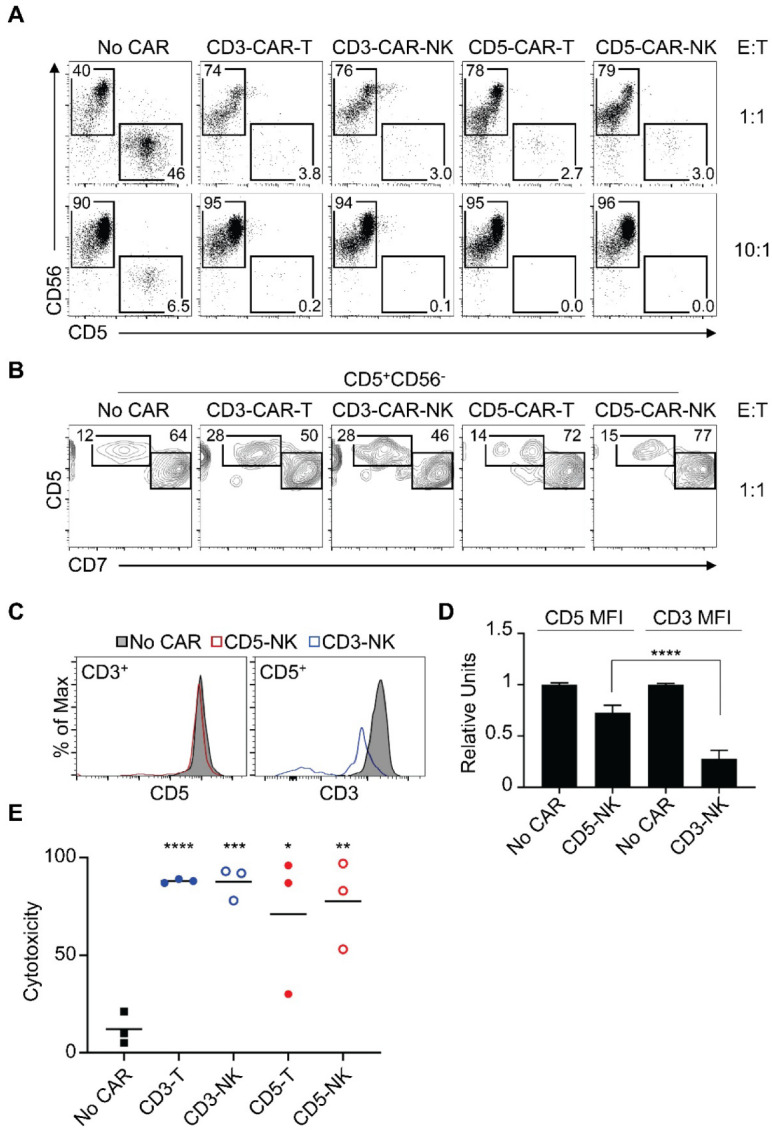
CD3 and CD5 CARs eliminate malignant T cells. (**A**) In vitro cytotoxic assay after incubation of the indicated CAR NK cells with T-cell lymphoma samples at the indicated effector-to-target ratios. This graph represents one of three independent experiments with three different patient samples. (**B**) FACS analysis of the phenotype of remaining target cells after coculture with the indicated CAR NK cells. (**C**) FACs analysis of cell surface levels of remaining primary T cells after coculture with CAR NK cells. Analysis of CD5 levels were performed on gated CD3+ cells and analysis of CD3 levels were performed on gated CD5+ cells. (**D**) Quantitation of cell surface levels represented in (**C**), from three independent experiments. To allow direct comparison of CD5 and CD3 MFI levels, relative MFI values were derived by dividing MFI levels of either CD5 or CD3 to the average of MFI levels in the No-CAR condition. (**E**) Quantitation of cytotoxicity represented in (**A**) in three patient samples. Each dot represents a patient sample. Cytotoxicity was analyzed as reduction in lymphoma cell numbers after coculture with effector NK cells. Unpaired *t*-test, *n* = 3. *p* < 0.05 (*), *p* < 0.01 (**), *p* < 0.001 (***), *p* < 0.0001 (****).

We next tested the cytotoxic activity of the CARs to eliminate tumor cells from three patients with peripheral T-cell lymphoma with significant blood and/or bone marrow involvement. For these experiments, we cocultured CAR-expressing NK cells with patient PBMCs or bone marrow and analyzed remaining cells by FACS. Based on previous phenotypic analysis of patient samples, remaining T cells were identified as CD5+ CD7+ and T-cell lymphoma cells as CD5+ CD7‒. We have also identified effector NK cells as CD56+ and B cells as CD19+ to serve as an internal negative control. Our results show that all CARs were highly effective to eliminate all T cells including lymphoma cells, shown as a reduction in CD56-CD5+ cells after coculture (Figure 3A). Cytotoxic activity was similarly observed on T cells (CD5+ CD7+) and lymphoma cells (CD5+ CD7‒), as remaining cells had a similar T cell: lymphoma proportion after treatment (Figure 3B). We quantitated the cytotoxic activity in a 1:1 effector-to-target ratio on lymphoma cells (CD5+ CD7‒) from three different patients and found that all CAR constructs were highly effective to eliminate lymphoma cells compared to NK cells not expressing a CAR (Figure 3E). 

We next measured the cell surface levels of target molecules in remaining T cells in the culture. To perform this analysis, we gated on CD56-CD3+ cells to analyze CD5 levels or on CD56-CD5+ cells to analyze CD3 levels. We observed a clear downregulation of CD3 levels after CD3-CAR-NK treatment, and only minimal downregulation of CD5 levels after CD5-CAR-NK treatment (Figure 3C). To quantitate these data from three patient samples, we derived relative mean fluorescent intensity (MFI) values of corresponding targets by dividing them to the average MFI of T cells cocultured with NK effector cells without CARs (No-CAR condition). Target T cells downregulated cell surface CD3 levels by approximately 75% and CD5 levels by approximately 25% after coculture with CD3-CAR-NK and CD5-CAR-NK, respectively. Downregulation of CD3 was significantly higher than that of CD5 (Figure 3D). 

### 3.4. Activity of the CAR Constructs In Vivo

**Figure 4:**
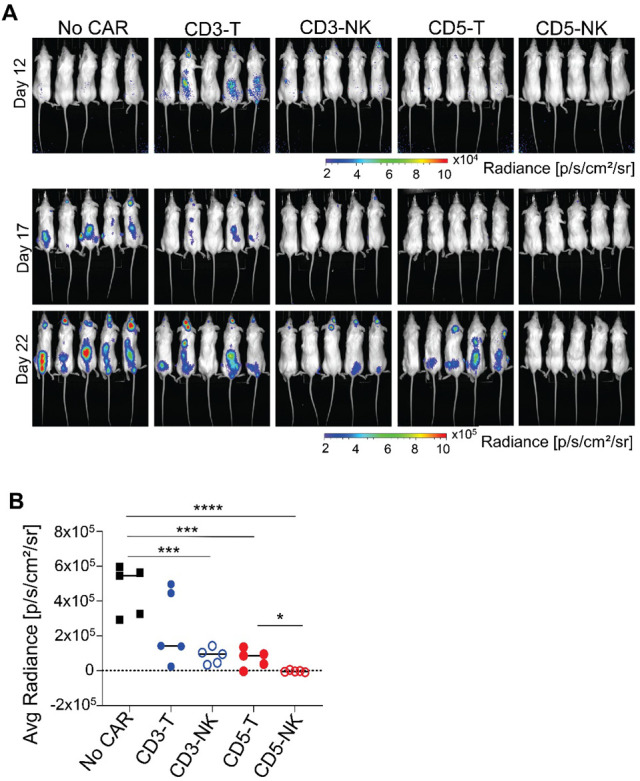
Elimination of Jurkat cells in a diffuse xenograft mouse model treated with CD3 and CD5 CAR−T and CAR−NK NK cells. (**A**) IVIS imaging of tumor xenografts in NSG mice at different time points after i.v. injection of 1 × 10^6^ Jurkat cells (Day 0) and NK cells expressing different CAR constructs at day 3 and 8. (**B**) Quantitative analysis of average radiance measured in different groups of mice injected with indicated CARs was compared to that of vector control NK-92 injected mice at 22 days post tumor injection. Each dot represents an individual mouse. Unpaired *t*-test, *n* = 5 per group, *p* < 0.05 (*), *p* < 0.001 (***), *p* < 0.0001 (****). This experiment is representative of two independent experiments with similar results.

We next evaluated if the increased activity of the CAR-NK framework would lead to a more efficient antitumor activity in vivo using a diffuse xenograft model. NSG mice were injected intravenously with Jurkat cells constitutively expressing luciferase, and three days later mice were injected with NK cells expressing different CARs. Analysis of luminescence by IVIS imaging showed that CD3-CAR-T NK cells had some antitumor activity that did not reach significance, and that CD3-CAR-NK NK cells had a significantly reduced tumor burden, but the tumor was still present 22 days after treatment. CD5-CAR-T NK cells significantly reduced but did not eliminate the tumor, while the antitumor activity of CD5-CAR-NK NK cells was robust, leading to elimination of the tumor (Figure 4A,B). These results show that CD5 is a better target than CD3 for T-cell lymphomas and that a NK-specific CAR framework, when expressed in NK effector cells, is more efficient to target tumors in vivo in correlation with its increased cytotoxic activity. 

## 4. Discussion

Several limitations exist for the clinical application of CAR-T cells as a therapy for T-cell malignancies. Expression of the target antigen in CAR-T cells would lead to a fratricide effect in which CAR-T cells would be target of their own attack. This prevents efficient amplification of CAR-T cells in culture before introduction to the patient. In addition, expression of the target molecule in healthy T cells would lead to T-cell aplasia and long-term immunodeficiency associated with CAR-T-cell persistence.

These limitations may be overcome by the use of NK cells as effector cells instead of T cells to express a CAR, as NK cells do not express many T-lineage markers that could be used for targeted therapy. NK cells may be allogeneic as they would not react to the recipient HLA and would not cause graft vs. host disease. Use of allogeneic donor cells would also eliminate the risk of contamination of the CAR product with tumor cells. In addition, NK cells are short-lived, so they would not persist in the host and would not cause long-term T-cell aplasia when targeting T-cell lineage molecules [15].

Introduction of CAR into NK cells has been performed for several tumor types, and their efficacy to eliminate B-cell leukemias had been shown in the clinic [16]. However, the same CAR framework that is used to generate CAR-T cells is commonly used for NK cells, which might not be the most efficient approach. A CAR-NK framework with higher in vitro activity when expressed by NK cells was first described using an antimesothelin-CAR reacting to glioblastoma cells [14]. 

In this work we performed a comparative analysis of the activity of CARs recognizing two established targets for T-cell lymphoma, CD3 and CD5 using a CAR-T or a CAR-NK framework. The scFv recognizing CD3 was derived from the sequence of the antibody OKT3 and has been described [6], and the scFv recognizing CD5 was derived from the humanized antibody H65 [3]. The CAR-T framework is composed of a transmembrane and co-stimulatory domain derived from CD28 and CD3ζ, and the CAR-NK framework is composed of a transmembrane domain of NKG2D, a costimulatory domain of 2B4 and CD3ζ.

NK92 cells overexpressing a CD3-CAR containing the costimulatory domains of 4-1BB and CD28 had shown cytotoxic activity against peripheral T-cell lymphoma and T-ALL cells in vitro and reduction in tumor burden in a xenograft model [17]. The same group later reported that NK92 cells overexpressing a CD5-CAR with 4-1BB and CD28 costimulatory domains were efficient to eliminate T-ALL cells [18].

By direct comparison of the activities of CD3-CAR and CD5-CAR in a CAR-T and CAR-NK framework, we observed that the activity of NK cells expressing the CAR-NK framework was superior to that of cells expressing the CAR-T framework when using effector NK cells. This was observed as increased degranulation and cytokine secretion by CD3-CAR and CD5-CAR. However, the CAR-NK framework only increased the cytotoxic activity in vitro of the CD5-CAR and not that of the CD3-CAR. This was due to the already high cytotoxicity levels of the CD3-CAR in the CAR-T framework, as the advantage of the CAR-NK framework could be observed when the number of effector cells was reduced to suboptimal levels.

2B4/CD244 is a member of the signaling lymphocyte activation molecule (SLAM) family that is expressed by all NK cells, γδT cells, monocytes and memory CD8 T cells [19]. Interaction with CD48, which is expressed in all hematological cells, leads to its activation [20]. Although its function has been reported as inhibitory or stimulatory depending on the cell type, it has been shown to be essential for the cytotoxicity of NK cells as disruption of its downstream signal by SAP knockdown in NK cells abrogated cytotoxicity [21]. As competing stimulatory and inhibitory signals determine the activation of NK cells, and in the absence of SAP the inhibitory molecules SHP1/SHIP2 bind to 2B4 [22], it is postulated that 2B4 engagement in NK cells tips the balance towards activation and increases cytotoxicity.

Similarly, NKG2D is an activating receptor in NK cells that has no intracellular signaling domain [23]. The NKG2D transmembrane domain interacts with DAP10 which activates downstream signals, leading to increased cytotoxicity [24]. 

Previous work has shown that incorporation of 2B4 as a costimulatory domain increases the activating potential of an anti-CD5-CAR in NK92 cells. Our CAR-NK framework uses a transmembrane NKG2D molecule in addition to 2B4 and it remains to be tested if it is more active than the described CAR, which lacks transmembrane NKG2D [25]. The anti-CD5 scFv sequence used in our CD5-CAR also differs from this previous work as the authors used the sequence of an anti-CD5 antibody they generated in their institute.

We observed that although CD3-CAR in the CAR-T-cell framework had higher in vitro activity than CD5-CAR, they were less active to reduce the tumor burden in a xenograft model, and this correlated with reduced CD3 cell surface levels after CAR treatment.

Loss of target antigen expression has been characterized as a main mechanism of relapse after CAR-T-cell treatment of B-cell malignancies [26]. However, selection of already present malignant B cells expressing truncated forms of the CD19 antigen by CAR-T cells has been described as the mechanism of escape [27]. Target antigen downregulation by persistence of CAR-T cells, not related to mutations or transcriptional regulation, has also been shown to reduce the effectiveness of an anti-CD22-CAR for B-cell malignancies [28]. Therefore, it is possible that downregulation of cell surface CD3 levels after CAR treatment may negatively affect their efficacy in vivo. TCR engagement as well as anti-CD3 stimulation of primary T cells and cell lines leads to rapid internalization and long-lasting degradation of the TCR-CD3 complex [29], which may account for the reduction in cell surface CD3 levels that we observed after CD3-CAR treatment. On the contrary, it is expected that engagement of CD5 in lymphoma and T cells would not elicit activation of target T cells or such a downregulation mechanism. Although we did observe a reduction in CD5 levels after anti-CD5-CAR treatment, this was of a lower magnitude than the downregulation of CD3 levels. Our results therefore support a preferential use of CD5 as a target antigen for T-cell lymphomas.

Despite this, the advantage of the CAR-NK framework was observed in vivo in both CD3 and CD5 CAR constructs, as CD3-CAR in a CAR-NK framework significantly reduced the tumor burden and CD5-CAR in a CAR-NK framework completely eliminated the tumor.

Interestingly, the beneficial advantage of either CD5-CARs or the CAR-NK framework was not observed in peripheral T-cell lymphoma patient samples and T cells in vitro, and all constructs were similarly effective. It is possible that this discrepancy is due to the short timeframe of in vitro cytotoxicity (hours) versus the long timeframe (days) required to observe an antitumor effect in vivo.

Finally, although anti-CD3 antibody treatment is a potent immunosuppressant to revert allograft rejection, it may also lead to nonspecific activation of T cells and toxicity associated with a cytokine release syndrome [30]. This toxicity has not been reported for treatment with anti-CD5 antibodies. Therefore, CD5-CARs may represent a safer choice.

## 5. Conclusions

To investigate the best CAR framework to target T-cell lymphomas by NK effector cells, we compared the activity of a CAR-T and a CAR-NK framework to target CD3 and CD5 on T cells. We observed that the CAR-NK framework conferred superior activity to the CAR-T-cell framework when targeting both CD3 and CD5 in vitro and in vivo. We also observed that although CD3-CAR constructs were highly efficient to target lymphoma in vitro, they were inefficient to target xenograft tumors, in correlation with strong downregulation of the target antigen after coculture with CD3-CAR NK cells. In conclusion, our data establish that a CAR-NK framework is superior to a CAR-T framework when using NK effector cells, and that CD5 is a better target antigen than CD3 for CAR treatment of T-cell malignancies.

## Supplementary Materials

The following supporting information can be downloaded at: https://www.mdpi.com/article/10.3390/cancers14030524/s1, Figure S1: CAR NK cells specifically target cells expressing the antigen of interest but not the control cell line.
