# Supplementary material for: Increased Activity of a NK-Specific CAR-NK Framework Targeting CD3 and CD5 for T-Cell Leukemias
Source: Cancers (Basel). 2022 Jan 21;14(3):524. doi: 10.3390/cancers14030524 (PMC8833462; doi:10.3390/cancers14030524)
Supplement: Supplementary file 1 [file cancers-14-00524-s001.zip › cancers-1536148-supplementary.pdf]

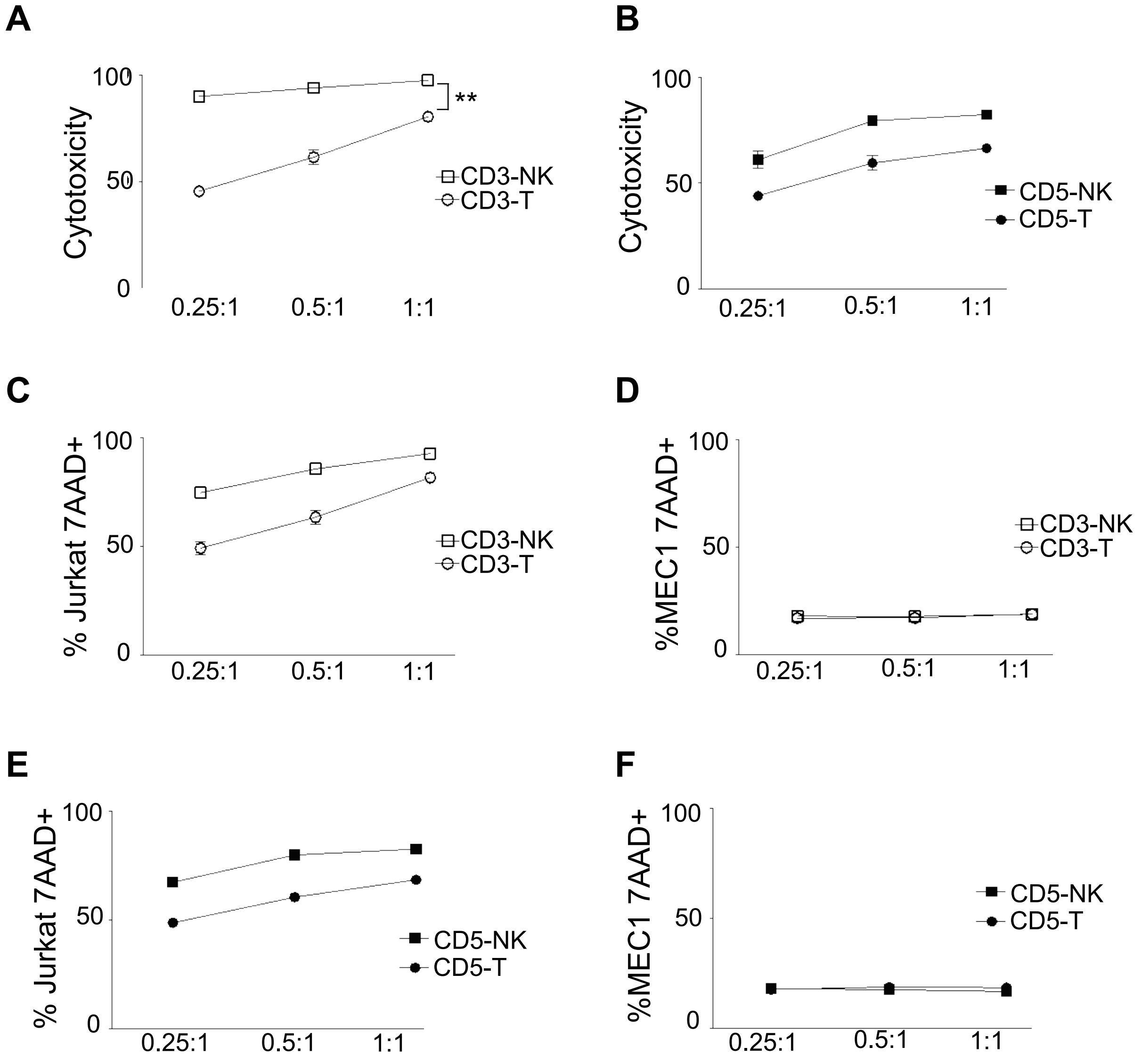

Supplemental Figure S1. CAR NK cells specifically target cells expressing the antigen of interest but not the control cell line. In vitro cytotoxic killing assay after incubation of indicated CAR NK effector cells with target cells (Jurkat) and control cell line (MEC1) for 4 hours at the indicated effector: target ratios. Percentage of specific cytotoxicity (A and B) calculated as explained in Materials and Methods, Percentage of 7AAD positive Jurkat (C, E) and MEC1 (D, F) cells. These graphs represent one of two independent experiments. Unpaired t-test, n = 2, $p<0.01$ (**).
